# Assessing the oncolytic potential of rotavirus on mouse myeloma cell line Sp2/0-Ag14

**DOI:** 10.7705/biomedica.4916

**Published:** 2020-06-30

**Authors:** Rafael A. Guerrero, Carlos A. Guerrero, Fanny Guzmán, Orlando Acosta

**Affiliations:** 1 Departamento de Ciencias Fisiológicas, Facultad de Medicina, Universidad Nacional de Colombia, Bogotá, D.C., Colombia Universidad Nacional de Colombia Departamento de Ciencias Fisiológicas, Facultad de Medicina Universidad Nacional de Colombia BogotáD.C Colombia; 2 Núcleo de Biotecnología Curauma, Pontificia Universidad Católica de Valparaíso, Valparaíso, Chile Pontificia Universidad Católica de Valparaíso Núcleo de Biotecnología Curauma Pontificia Universidad Católica de Valparaíso Valparaíso Chile

**Keywords:** Oncolytic viruses, rotavirus infections, neoplasms/therapy, virus oncolíticos, infecciones por rotavirus, neoplasias/terapia

## Abstract

**Introduction:**

Cancer is the second leading cause of death in the United States, surpassed only by cardiovascular disease. However, cancer has now overtaken cardiovascular disease as the main cause of death in 12 countries in Western Europe. The burden of cancer is posing a major challenge to health care systems worldwide and demanding improvements in methods for cancer prevention, diagnosis, and treatment. Alternative and complementary strategies for orthodox surgery, radiotherapy, and chemotherapy need to be developed.

**Objective:**

To determine the oncolytic potential of tumor cell-adapted rotavirus in terms of their ability to infect and lysate murine myeloma Sp2/0-Ag14 cells.

**Materials and methods:**

We inoculated rotaviruses Wt1-5, WWM, TRUYO, ECwt-O, and WTEW in Sp2/0-Ag14 cells and we examined their infectious effects by immunocytochemistry, immunofluorescence, flow cytometry, and DNA fragmentation assays.

**Results:**

Rotavirus infection involved the participation of some heat shock proteins, of protein disulfide isomerase (PDI), and integrin β3. We detected the accumulation of viral antigens within the virus-inoculated cells and in the culture medium in all the rotavirus isolates examined. The rotavirus-induced cell death mechanism in Sp2/0-Ag14 cells involved changes in cell membrane permeability, chromatin condensation, and DNA fragmentation, which were compatible with cytotoxicity and apoptosis.

**Conclusions:**

The ability of the rotavirus isolates Wt1-5, WWM, TRUYO, ECwt-O, and WTEW to infect and cause cell death of Sp2/0-Ag14 cells through mechanisms that are compatible with virus-induced apoptosis makes them potential candidates as oncolytic agents.

## Resumen

**Introducción.:**

El cáncer es la segunda causa de muerte en los Estados Unidos, solamente superado por la enfermedad cardiovascular. Sin embargo, el cáncer aventaja a la enfermedad cardiovascular como primera causa de muerte en doce países de Europa occidental. Se requieren mejores métodos de prevención, diagnóstico y tratamiento para afrontar el gran desafío que el cáncer representa mundialmente para los sistemas de salud, y se necesita desarrollar estrategias alternativas y complementarias a la cirugía, la radioterapia y la quimioterapia convencionales.

**Objetivo.:**

Evaluar el potencial oncolítico de rotavirus adaptados a células tumorales por su capacidad para infectar y lisar células Sp2/0-Ag14 de mieloma de ratón.

**Materiales and métodos.:**

Los aislamientos de rotavirus Wt1-5, WWM, TRUYO, ECwt-O y WTEW se inocularon en células Sp2/0-Ag14 y se examinaron sus efectos infecciosos mediante inmunocitoquímica, inmunofluorescencia, citometría de flujo y ensayos de fragmentación del ADN.

**Resultados.:**

La infección con los rotavirus Wt1-5, WWM, TRUYO, ECwt-O y WTEW implicó la participación de algunas proteínas de choque térmico, la proteína disulfuro isomerasa y la integrina β3. La acumulación de antígenos virales intracelulares y extracelulares se detectó en todos los virus utilizados. Los mecanismos de muerte inducidos por los rotavirus en células Sp2/0-Ag14 indujeron cambios en la permeabilidad de la membrana celular, la condensación de cromatina y la fragmentación de ADN, los cuales fueron compatibles con citotoxicidad y apoptosis.

**Conclusiones.:**

La capacidad de los rotavirus estudiados para infectar y causar la muerte de células Sp2/0-Ag14 mediante mecanismos compatibles con la apoptosis inducida viralmente los convierte en candidatos potenciales para ser utilizados como agentes oncolíticos.

Cancer is the second leading cause of death in the United States, surpassed only by cardiovascular disease ^1^. However, cancer has now overtaken cardiovascular disease as the main cause of death in twelve countries located in Western Europe ^2^. The burden of cancer is posing a major challenge to health-care systems worldwide and this demands an improvement of tools for cancer prevention, diagnosis, and treatment. Alternative and complementary strategies for orthodox surgery, radiation therapy, and chemotherapy need to be developed.

Advances in molecular biology research, including new information about tumoral cells, have a significant impact on the development of methods for treating cancer such as small interfering RNA (siRNA) ^3^, microRNA (miRNA) ^4^, cell signaling-based chemotherapy ^5^, and virotherapy ^6^, among others.

What we know about cell signaling pathways of tumoral cells suggests that tumor cell-lineage diversity converges into common molecular pathways regulating growth and differentiation while some atypical lineages behave in a unique way ^7^. However, tumors originating from the same cell type but different individuals can behave differently because they start from different genetic backgrounds and have a different evolution process ^8^. This heterogeneity suggests an individualized approach for the management of each tumor type ^9^^,^^10^.

In this context, viral oncolytic therapy as an anticancer strategy for the treatment of some tumors has been introduced to the extent that the knowledge about molecular mechanisms of carcinogenesis and virus infection has undergone significant progress ^11^^,^^12^. This therapy is based on viral particles that have been selected or genetically modified to proliferate specifically in tumor cells leading to their death ^13^. The oncolytic virus-based therapy promotes anti-tumor responses involving selective tumor cell killing and induction of systemic anti-tumor immunity ^14^.

Genetic mutations occurring in tumor cells make them more susceptible to oncolytic viruses whose tropism for these neoplastic cells depends on their transcription machinery and signaling pathways ^15^^,^^16^. The oncolytic virus entry into the tumor cell depends on the cell surface receptor-mediated binding of the virus to target cells before penetration. Since these receptors are absent in normal cells but over-expressed in some tumor cells, besides being oncolytic virus-specific, they allow oncolytic viruses to be an efficient and safe tool for cancer treatment.

Owing to the ability of oncolytic viruses for replicating within tumor cells, they exhibit unique pharmacokinetic characteristics that differentiate them from conventional cancer treatment. Oncolytic viruses can be genetically modified and redesigned relatively easily to introduce in their genome toxin-encoding genes that are harmful to tumor cells or genes encoding for immunostimulant products ^17^. However, there is no oncolytic virus or conventional therapeutic strategy good enough for treating all tumors since their tissues have complex biology where individual cells within the same tumor type can have different biochemistry ^18^^,^^19^.

It has been well documented that heat shock proteins (HSPs) are involved in several crucial events associated with tumor development including regulation of cell cycle progression ^20^^-^^22^, control of apoptotic pathways ^22^^,^^23^, and immune surveillance against cancer ^24^^,^^25^. Heat shock protein overexpression is observed in human, murine, and canine neoplasms indicating that HSP play a role in carcinogenesis and metastasis of brain, lung, breast, and prostate cancers, as well as in sarcomas and some lymphomas ^26^. The expression of Hsc70 ^27^, PDI ^28^, integrin β3 ^29^, and several heat shock proteins has been associated with cell malignancy ^26^. Hsc70 ^30^ and PDI ^31^ are either expressed at very low levels or not at all on the cell surface of normal cells while tumor cells express them and several HSP at relatively high levels ^32^.

Several studies have shown rotavirus tropism in cells expressing Hsc70 ^33^, PDI ^34^, and integrins such as αVβ3 ^35^. Recently, it was described how some reassorted rotavirus isolates can successfully replicate in some tumor cell lines ^36^.

In the present study, we determined the oncolytic potential of rotaviruses using a model consisting of cultured murine myeloma cells. Our results demonstrate that the reassortant rotaviruses WTEW, Wt1-5, TRUY, and WWM, as well as ECwt-O, were able to successfully infect, replicate, encapsidate, and lyse murine myeloma Sp2/0-Ag14 cells. Rotavirus-induced changes in infected cells were compatible with toxicity and apoptosis. The results allowed suggesting that Hsp90, Hsp70, Hsc70, PDI, and integrin β3 participate during rotavirus entry into the target cell and that rotavirus infection results in expression changes of cellular proteins Hsp90, Hsp70, and Hsc70.

## Materials and methods

### Cell lines

Sp2/0-Ag14 cells (mouse B cell myeloma) were obtained from American Type Culture Collection^™^ (ATCC-CRL-1581). L929 cells (L cell, L-929, a derivative of strain L, ATCC1 CCL-1) were kindly donated by C. Parra of the *Facultad de Medicina* at *Universidad Nacional de Colombia*. Peripheral blood mononuclear cells (PBMC) were isolated from a human donor using Ficoll-Paque^™^.

The present study had prior approval of the Ethics Committee of the *Facultad de Medicina* at *Universidad Nacional de Colombia*.

All cell lines were cultured using Corning cell culture flasks (Sigma-Aldrich, St. Louis, MO, USA) in Dulbecco´s Modified Eagle Medium (DMEM) or RPMI 1640 (Sigma-Aldrich, St. Louis, MO, USA) supplemented with 10% fetal bovine serum (FBS) (Eurobio, Les Ulis, France) and 100 μg/ml streptomycin and penicillin (Eurobio, Les Ulis, France).

All cells were cultured at 37 °C in a humidified atmosphere with 5% CO_2_. The culture medium was changed every 3 days. To assess population doubling time, the culture medium was discarded and the cells washed 3 times with PBS containing 0.03% EDTA before incubation with PBS-EDTA containing trypsin (1 µg/ml) for 5 min at 37 °C. The detached cells were immediately dispersed in culture medium supplemented with FBS and subcultured in a new flask for estimating PDT.

### A*ntibodies and reagents*

Goat antibodies against Hsp90 (SC-1055), Hsp70 (SC-1060), Hsp60 (SC-1052), Hsp40 (SC-1801), Hsc70 (SC-1059), integrin β3 (SC-6626), PDI (SC-17222), Cox-2 (SC-1747), and cleaved PARP-1 (SC-56196) were obtained from Santa Cruz Biotechnology Inc. (Santa Cruz, CA, USA). Rabbit antibodies against NF-kB p65 (phospho S536) (ab86299) were purchased from Cambridge Science Park (Cambridge, UK). Donkey anti-goat Alexa Fluor 594-conjugated secondary antibodies (SC- 362275) and donkey anti- rabbit-Alexa Fluor 594- conjugated secondary antibodies (SC-362281) were obtained from Santa Cruz Biotechnology Inc. (Santa Cruz, CA, USA). Donkey anti-goat or anti-rabbit antibodies conjugated with FITC (SC-362255 and SC-362261, respectively) or HRP (SC-2020 and SC-2313, respectively) were also obtained from Santa Cruz Biotechnology Inc. 7-aminoactinomycin D (7-AAD), propidium iodide, and 4,’6-diamidino-2-phenylindole (DAPI) were purchased from Invitrogen (Carlsbad, CA, USA). Annexin V-Alexa 568 kit, Apoptotic DNA- Ladder Kit, In Situ Cell Death Detection kit, and poly(ADP-ribose) polymerase (PARP) were obtained from Roche Laboratories, Inc. (Nutley, NJ, USA), and Hoechst 33342 from Thermo Scientific (Waltham, MA, USA).

### Rotavirus isolates

Rotavirus isolates Wt1-5, WWM, TRUYO, ECwt-O, and WTEW, selected as previously reported ^36^, were trypsin-activated (10 mg/ml) and separately inoculated (MOI of 2) in Sp2/0-Ag14 cells (1 x 10^7^) in DMEM (10 ml) without FBS. The cells were cultured at 37 °C until their lysis (about 24 h). The infection cycle was repeated four times in new cells and subsequent infections were performed with virus isolates without trypsin treatment.

### Synthetic peptides

Synthetic peptides derived from HSP were prepared using a solid- phase method as previously described ^37^. The peptide sequences were as follows: Hsp90 (620-RDNSTMGYMAAKKHLEINPDHS-641); 

Hsp70 (374) (705-QIQQYMKIISSFKNKEDQYDHLD-727);

Hsp70 (375) (646-NSFTLKLEDTENWLYEDGDQPKQ-668);

Hsp70 (376) (741-AMEWMNNKLNLQNKQSLTMDP-761); 

Hsp60 (393-RLAKLSDGVAVLKVGGTSDVEVN-415); Hsp40 

 (251-GSDVIYPARISLREALCGCTVNV-273).

### Antisera preparation

New Zealand rabbits were subcutaneously immunized with 1 ml of a Freund’s complete adjuvant emulsion containing the respective specific HSP peptides (0.5 mg/ml) mixed with FIS (FISEAAIIHVLHSR) peptide (0.5 mg/ ml) as an immunomodulatory agent ^38^. The same amount of each antigen emulsified in Freund’s incomplete adjuvant was inoculated to rabbits 20 and 40 days later. Bleeding of rabbits was performed on day 60 after the first injection. Sera containing sodium azide (0.04%) and diluted two-fold with glycerol were kept at -20 °C until use. Control pre-immune sera were collected before immunization.

All procedures involving rabbits were performed according to the Ethics Committee’s approval.

### Cell infection

The culture medium was removed from cells (Sp2/0-Ag14, L929 or PBMCs) and DMEM without FBS was added to wash twice the cells. Sp2/0- Ag14 cells (1.5 x 10^6^) were incubated in DMEM without FBS (1.0 ml) in 12-well plastic plates at 37 °C with 5% CO_2_ and then separately infected with cesium chloride-purified isolates Wt1-5, ECwt-O, TRUYO, WWM, or WTEW at an MOI of 0.8 each ^39^.

Cells were harvested at 0, 2, 4, 6, 8, 10, 12, and 24 hpi and fixed with 4% paraformaldehyde in PBS for 30 min at room temperature. In the case of non- tumoral cells L929 and PBMCs, increasing MOIs (0.5, 1, 1.5, 2, 3, and 6) were tested. The fixed cells were washed twice with PBS for 5 min each wash and resuspended in PBS containing 0.02% sodium azide before storing at 4 °C until use. Infection was assessed by immunocytochemistry as indicated below.

The effect of antibodies against HSP, PDI, and integrin β3 on rotavirus infection was tested by incubation of Sp2/0-Ag14 cells with hyperimmune antiserum against Hsp90, Hsp70, Hsp60, Hsp40, Hsc70, PDI, or integrin β3 diluted (1:25, 1:50, 1:100, 1:200, 1:400 or 1:800) in PBS containing 1% BSA. The antisera were generated in rabbits that had been immunized with human synthetic peptides derived from active sites of Hsp90, Hsp70, Hsp60, and Hsp40, or complete rHsc70 ^40^ and integrin β3 isolated from platelets.

Cells were incubated for 1 h at 37 °C, washed 3 times with PBS, placed on ice for 15 min, and then inoculated with rotavirus isolates (MOI of 0.8) and incubated for 45 min at 4 °C. The cells were further incubated at 37 °C for 12 h and subjected to immunochemistry assay for the detection of rotavirus structural antigens. The percentage of infection was determined in the absence of antibody treatment. Cells treated with the unrelated antibodies against potato virus Y (Agdia AUG96 Lot # 0427) and then infected with rotavirus isolates were used as a control. Hyperimmune antisera were tested for the absence of anti- rotavirus antibodies using immunofluorescence, Western blotting, and ELISA.

### Immunocytochemistry

Fixed Sp2/0-Ag14 cells were applied onto coverslips previously washed with xylol, dried at 50 °C for about 30 min, and slightly flamed. The cells were permeabilized with 0.5% Triton X-100 for 5 min at RT and washed twice with PBS for 5 min each. Rabbit antibodies (1:2,000) against rotavirus structural proteins (triple layer particles) and non-structural proteins (recombinant versions of NSP-4 and NSP-5) were added and incubated for 1 h at 37 °C. After washing twice with PBS, donkey anti-rabbit HRP-conjugated secondary antibody (0.133 µg/ml) was added and incubated for 1 h at 37 °C. After washing with PBS twice, the reaction was visualized with 3-amino-9-ethylcarbazole (AEC) (0.64 mg/ml) in 50 mM sodium acetate buffer, pH 5.5, containing 0.03% H_2_O_2_.

Rotavirus-infected Sp2/0-Ag14 cells treated with unrelated isotype- matched antibodies and non-infected Sp2/0-Ag14 cells were used as a control. The percentage of viral antigen-positive cells was determined and representative photographs were taken. The viral titer was estimated from the number of rotavirus-positive cells after 12 hpi taking into account the serial dilutions of the concentrated viral stock.

### Immunofluorescence

An immunofluorescence assay was conducted to assess expression changes for Hsp40, HSP60, HSP70, HSP90, Hsc70, integrin β3, and PDI. Cells were infected with Wt1-5, ECwt-O, TRUYO, WWM, or WTEW as indicated above. Cells were fixed, dried, and permeabilized as indicated for immunochemistry. Cells were treated with 50 mM NH4Cl and 100 mM glycine in PBS for 30 min at RT to quench auto-immunofluorescence. Cells were stained with DAPI (0.1 µg/ml) for 30 min in a dark humid chamber at 37 °C and then washed twice with PBS for 5 min each time.

Cells were incubated with goat antibodies (0.2 µg/ml in PBS containing 1% BSA) against HSP40, HSP60, HSP70, HSP90, Hsc70, integrin β3, or PDI for 1 h in a dark humid chamber at 37 °C. After two washes with PBS, donkey anti- goat antibodies conjugated to Alexa Fluor 568 (5.7 µg/ml) in 1% BSA in PBS were added and maintained for 1 h in a dark humid chamber at 37 °C. Coverslips were washed twice with PBS and mounted inverted on glass microscope slides using 70% glycerol in PBS and resin. Non-infected cells subjected to the same procedures were used as a control.

### Flow cytometry and epifluorescence

Expression of Hsp40, HSP60, HSP70, HSP90, Hsc70, integrin β3, and PDI “on cell surface” was assessed by flow cytometry and epifluorescence. Cells at the logarithmic growth phase were collected, fixed with 4% glutaraldehyde, and washed twice with PBS before the addition of goat antibodies (0.2 µg/ml) to Hsp40, HSP60, HSP70, HSP90, Hsc70, integrin β3, or PDI. Alternatively, rabbit hyperimmune antisera (1:2,000) raised against synthetic peptides (20 aa) spanning the active site of HSP40, HSP60, HSP70, and HSP90, or against a recombinant version of complete Hsc70 and PDI, or integrin β3 purified from platelets, were also used as primary antibodies.

Following incubation for 1 h at 37 °C, cells were washed 3 times with PBS and incubated with secondary donkey anti-goat or anti-rabbit antibodies conjugated to FITC (0.88 µg/ml) in PBS containing 1% BSA. Cell analysis was performed using a Becton Dickinson FACS Canto II^™^ flow cytometer. Cells were also analyzed by epifluorescence using a Nikon C1 Eclipse^™^ confocal laser microscope equipped with the Acquisition Software Nikon EZ-C1™, version 3.90.

### Cytotoxic, genotoxic, and apoptotic effects

To determine cell viability following virus infection, cells collected at the logarithmic phase of growth were tested for their viability using the trypan blue exclusion test. Cells were then infected with rotavirus isolates Wt1-5, ECwt-O, TRUYO, WWM, or WTEW at MOI of 0.8 and cell viability recorded at 0, 2, 4, 8, 12, and 24 h after virus infection. Viability of L-929 cell monolayers was observed in an inverted microscope (Euromex) whereas that of Sp2/O- Ag14 cell suspensions was observed using a Neubauer chamber and a conventional microscope.

Genotoxic damage induced by rotavirus infection was assessed by the detection of poly (ADP-ribose) polymerase-1 (PARP-1) as indicative of the repair process of fragmented DNA. Procedures were conducted following the manufacturer’s instructions. Briefly, cells fixed on coverslips were washed twice with PBS-T (PBS containing 1% BSA and 1% Tween 20) before the addition of cleaved PARP-1 antibody (194C1439) (0.2 μg/ml, SC 56196) in PBS-T and incubation for 1 h at RT. Cells were washed twice with PBS-T and incubated with goat anti-mouse IgG conjugated to FITC (0.88 µg/ml) for 30 min at 4 °C. Coverslips were washed twice with PBS and mounted inverted onto glass slides using glycerol and resin as indicated above.

*In situ* DNA fragmentation in Sp2/0-Ag14-Ag14 cells separately infected (MOI of 0.8) with the different rotavirus isolates indicated above was also assessed using TUNEL assay (Invitrogen). Infected cells (1.5 x10^6^) were harvested after 12 h incubation at 37 °C and fixed with 4% of paraformaldehyde in PBS, pH 7.4, freshly prepared. The samples were washed 3 times in PBS and adjusted to 2 x 10^7^ cells/ml. The cells were resuspended in 100 μl/well of permeabilization solution (0.1% Triton X-100 in 0.1% sodium citrate, pH 7.0, freshly prepared) for 2 min on ice (2-8 °C) and then rinsed twice with PBS. The cells were placed onto coverslips and dried at 50 °C for 1 h before adding 50 ul of TUNEL reaction mixture. The coverslips were incubated in a humidified atmosphere for 60 min at 37 °C in the dark. After this incubation, the cells were rinsed three times with PBS. The samples were observed directly under a fluorescence microscope using an excitation wavelength in the range of 450-500 nm. Emission was recorded in the range of 515-565 nm. Non-infected and H_2_O_2_-treated cells were used as control.

Early apoptotic signals were assessed in Sp2/0-Ag14 cells that had separately been infected with the different rotavirus isolates (MOI of 0.8). Non- infected or H_2_O -treated cells were used as control. After 12 h of culture, cells (1 x 10^6^) were harvested and washed twice with PBS before suspension and incubation for 15 min at RT in 100 ml HEPES buffer, pH 7.4, containing 140 mM NaCl, 5 mM CaCl_2_, and Annexin V-Alexa Fluor 568^™^ (Roche) (20 µl/ml). Cellular membrane integrity was tested for its permeability to 7-AAD in rotavirus infected cells (MOI of 0.8) that had been incubated for 12 h at 37 °C. Cells (1x 106) were washed twice with PBS, collected by centrifugation (600*g*), and suspended in 1ml PBS containing 0.3 mM CaCl2, 0.3 mM MgCl2, 2% BSA, and 1 mg/ml 7-AAD for 20 min at 4 °C in the darkness. Cells were washed twice with PBS before observation using a fluorescence microscope.

Apoptotic effects in terms of nuclear condensation and fragmentation were estimated using Hoechst 33342/propidium iodide staining. After infection with rotavirus isolates, cells were collected at 2, 4, 6, 8, 10, 12, 24, and 36 hpi, fixed with 4% paraformaldehyde, and washed twice with PBS. Cells (1 x 10^6^) were suspended in PBS (100 µl) containing 0.02% sodium azide 0.25 ug/ml (Hoechst), 0.20 µg/ml and propidium iodide for 10 min at room temperature. Coverslips were washed twice with PBS and mounted inverted onto glass slides using glycerol and resin as indicated above.

Apoptotic effects were further analyzed using Apoptotic DNA-Ladder Kit^™^ (Roche) and following the manufacturer’s instructions. Briefly, cells (1 x 10^7^) were infected with rotavirus isolates at a MOI of 2 and incubated for 12 h at 37 °C; they were then harvested and added with 0.5 mM PMSF before storage at -20 °C. Cells (2 x 10^6^) in PBS (200 µl) were mixed with lysis buffer (200 µl) (6 M guanidine-HCl, 10 mM urea, 10 mM Tris-HCl, pH 4.4, 20% Triton X-100) for 10 min at RT. DNA was eluted twice from the filter using preheated (70 °C) elution buffer (200 µl) (10 mM Tris-HCl, pH 8.5). After elution buffer addition, the filter tube was centrifuged at 6.200*g* for 1 min and the eluted DNA stored at -20 °C.

DNA quantity and purity were assessed using a NanoDrop 2000c (Thermo Scientific). DNA from non-infected cells was used as a negative control. Cells treated with H_2_O were used as a positive control. DNA samples were analyzed by electrophoresis on a 1% agarose gel at 5 V/cm for 1.5 h. Gels were stained with SYBR-Safe DNA gel stain^™^ (Thermo Scientific, Waltham, MA, USA) diluted 1:10.000 in TBE buffer (89 mM tris-borate, pH 8.3, and 2 mM EDTA), visualized with UV excitation, and photographed using a 10-megapixel Canon camera^™^.

All fluorescence analyses were conducted using a Nikon C1 confocal laser scanning microscope. Images were captured using EZ-C1 Nikon software. DAPI staining was visualized using laser excitation at 408 nm and detection at 450/35 nm. Fluorescence from Alexa Fluor 568 was observed using laser excitation at 543 nm and detection at 605/75 nm. Images were analyzed using the ImageJ 1.44p Java 1.6.0_20 (32-bit) software.

### ELISA

ELISA analyses were conducted as previously described ^36^. Briefly, Sp2/0-Ag14 cells were separately infected with the rotavirus isolates described above. Infected cells were harvested after incubation for 12 h at 37 °C and collected by centrifugation at 600*g* for 5 min. The supernatant was added with RIPA buffer (150 mM NaCl, 1% NP-40, 0.5% DOC, 0.1% SDS, 50 mM Tris-HCl, pH 8.0, final concentrations) and centrifuged at 10,000*g* for 10 min at 4 °C. The resultant supernatant was applied to ELISA plate wells coated with guinea pig polyclonal antibodies against rotavirus structural proteins and incubated for 1 h at 37 °C.

Plates were washed three times with washing buffer (PBS-T) (PBS containing 0.05% Tween 20) and incubated with rabbit polyclonal antibodies against rotavirus structural proteins. After PBS-T washing three times, plate wells were added with HRP-conjugated goat anti-rabbit IgG (0.08 µg/ml, Santa Cruz SC-2313) and incubated for 1 h at 37 °C. The reaction was revealed with OPD (o-phenylenediamine dihydrochloride) diluted in stable peroxide substrate buffer. Purified ECwt-O particles were used as a positive control whereas supernatants from non-infected cells were used as a negative control. ELISA plates were read at 492 nm on an FLx800 Multi-Detection microplate reader ^TM^ (Biotek) and the results were expressed as delta optical density (OD).

## Results

### Rotavirus infection of Sp2/0-Ag14 cells

Previous experiments demonstrated that after multiple passages in human tumor cell lines, tumor cell-adapted rotavirus isolates can be obtained ^36^. Rotavirus isolates TRUYO, WWM, and WTEW derived from different combinations of parental rotavirus strains, isolate Wt1-5 derived from a combination of several patient-derived rotavirus isolates, and multiple- passaged murine ECwt-O were able to successfully infect several human tumor cell lines ^36^. To gain further insight into the viral life cycle leading to tumor cell death, we used human tumor cell line Sp2/O-Ag14.

**Figure 1:**
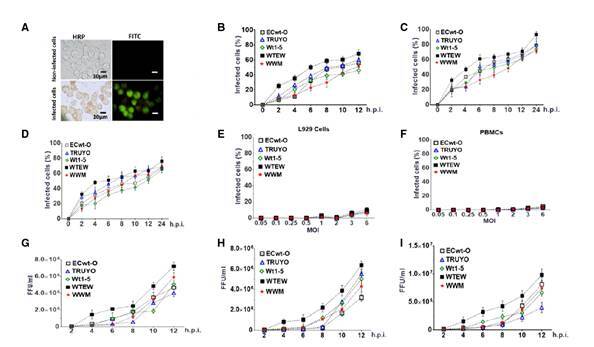
Infection of Sp2/0-Ag14 cells by rotavirus isolates and infectious virions present in supernatant from the culture medium. Sp2/0-Ag cells at the logarithmic growth phase were separately infected with previously trypsin- activated rotavirus isolates WTEW, WWM, Wt1-5, TRUYO, or ECwt-O at 0.8 MOI each. **A.** HRP immunochemistry staining of rotavirus structural antigens (reddish) at 12 hpi followed by FITC fluorescent staining (green). **B.** Sp2/0-Ag cells were separately infected with the rotavirus isolates mentioned above and cell aliquots taken at the indicated post-infection times. Infection was recorded as the percentage of cells being positive to rotavirus structural antigens using HRP immunochemistry assay. **C.** As in B, except that cells being positive to nonstructural antigen NSP 4 were recorded. **D.** As in B, except that cells being positive to non-structural antigen NSP5 were recorded. **E.** Control non-tumoral L929 cells were separately infected with the rotavirus isolates indicated above using increasing MOI. Infection is expressed as the percentage of cells being positive to rotavirus structural antigens at 12 hpi using HRP immunochemistry assay. **F.** As in E, except that control cells consisting of non-tumoral PBMCs were used instead of L926 cells. **G.** Sp2/0-Ag cells were infected as described in A and then aliquots of cells were collected every 2 h after infection with the indicated rotavirus isolates and subjected to centrifugation at 700*g*. The supernatant medium was collected and used to inoculate a fresh batch of Sp2/0-Ag cells, which were collected at 12 hpi and lysed; the infectious titers (FFU/ml) of lysates were determined by immunochemistry assay for the viral structural antigens. **H.** As in G, except that the non-structural antigen NSP4 was determined. **I.** As in G, except that the non-structural antigen NSP5 was determined. Infection is expressed as mean percentage ± SD from three independent experiments (n=3), each performed in duplicate.

Stock preparations of the rotavirus isolates Wt1-5, ECwt-O, TRUYO, WWM, or WTEW were diluted with MEM to reach inocula that were able to infect about 50% (approximately 0.8 MOI) of the Sp2/0-Ag14 cells according to the immunochemistry and immunofluorescence images obtained after 12 hpi at 37 °C (figure 1A). When the separately infected cells were tested every 2 hpi for the appearance of viral antigens, positive signals for these antigens were detected by immunochemistry assay as early as 2 hpi.

However, a progressive increase in the proportion of cells being positive to viral antigen was observed between 2 and 12 hpi. At the end of this period, the mean percentage of cells showing viral antigens was about 59%. The percentage of infected cells was determined in terms of structural (figure 1B) and non-structural proteins NSP4 (figure 1C) and NSP5 (figure 1D). Assays of infection with the same rotavirus isolates (MOI of 0.5, 1, 1.5, 2, 3, and 6) using non-tumor cells L929 (figure 1E) and human peripheral blood mononuclear cells (PBMCs) (figure 1F) showed negative results for viral antigens assessed by immunochemistry assay.

To assess the presence of extracellular viral antigens released into the culture medium, the 600*g* supernatant of Sp2/0-Ag14 cells that had been separately infected with rotavirus isolates Wt1-5, ECwt-O, TRUYO, WWM, or WTEW was tested for the presence of viral antigens using ELISA. The results showed that the infection produced viral antigens that were released into the culture medium and were easily detectable at 12 hpi for all the rotavirus isolates tested (data not shown).

To determine whether the virus inoculated was able to replicate and produce new mature and infectious virions, Sp2/0-Ag14 cells were separately infected with the same rotavirus isolates (MOI of 0.8). Lysates from infected cells taken every 2 hpi were able to successfully infect fresh cells as judged by the detection of rotavirus structural antigens (figure 1G) and non-structural antigens NSP4 (figure 1H) and NSP5 (figure 1I) by immunochemistry assay at 12 hpi. The results showed that all the rotavirus isolates studied generated infectious virions which were easily detected for their infectious capacity first at 4 hpi for WTEW and WT1-5, at 6 hpi for WWM and ECwt-O, and at 8 hpi for TRUYO (figure1G-I).

### Effects of rotavirus infection on cell viability and membrane integrity

**Figure 2:**
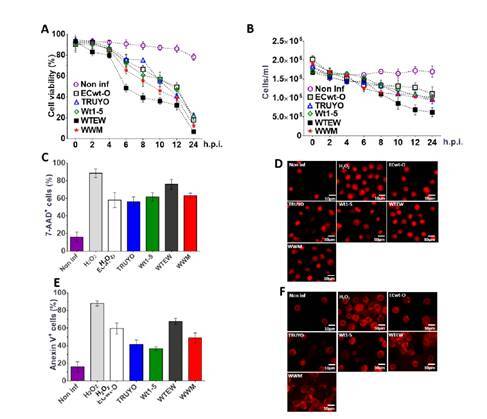
Effects of rotavirus infection on cell viability, membrane integrity, and nuclear morphology. Sp2/0-Ag cells at the logarithmic growth phase were separately infected with previously trypsin-activated rotavirus isolates WTEW, WWM, Wt1-5, TRUYO, or ECwt-O at 0.8 MOI each. Non-infected Sp2/=-Ag cells and 1 mM H_2_O_2_-treated Sp2/0-Ag cells were used as a control. **A.** The viability of cells collected at the indicated post-infection time was determined using the trypan blue exclusion test. **B.** The total number of the cells/ml remaining at the indicated post-infection times was determined using a Neubauer chamber. **C.** SP2/0-Ag cells infected with the indicated rotavirus isolates were stained with 7-AAD at 12 hpi. The percentage of cells being positive to 7-AAD fluorescence was determined for the rotavirus isolates indicated. **D.** Representative images of 7-AAD-stained cells at 12 hpi are shown. **E.** SP2/0-Ag cells infected with the indicated rotavirus isolates were stained with annexin V at 12 hpi. The percentage of cells being positive to annexin V was determined for each rotavirus isolate tested. **F.** Representative images of Annexin V-stained cells at 12 hpi are shown. Images were taken with a confocal microscope (Nikon-C1) and analyzed using the EZ-C1, ver. 3.90, software. Results are shown as mean percentages ± SD from two independent experiments performed in duplicate.

To determine the effects of rotavirus infection on cell viability and integrity of the cell membrane, Sp2/0-Ag14 cells were separately infected with the rotavirus isolates WT1-5, ECwt-O, TRUYO, WWM, or WTEW (MOI of 0.8). The results obtained by testing cells every 2 hpi until 12 hpi for their ability to exclude trypan blue revealed that cell viability started to decrease continuously from 6 hpi until reaching its lowest value (20%) at 12 hpi. In addition, the proportion of cells remaining viable at 24 hpi was less than 3% (figure 2A) when the percentage of observable cells was reduced to 45 to 50% compared with the number of control cells at 0 hpi (figure 2B). These results suggest that the decreased number of cells was caused by rotavirus- induced lysis of infected cells.

The exclusion test using 7-AAD, a membrane impermeant dye, confirmed that rotavirus-infected cells lost their ability to exclude this DNA-intercalating agent as 56 to 76% of infected cells depending on the virus isolated used showed fluorescent signals at 12 hpi. About 18% of non-infected control cells were positive to the 7-AAD test while about 90% of the H_2_O_2_-treated cells were found to be positive for this test (figure 2C). The pattern of fluorescent signals in infected cells was similar to that of control cells treated with H_2_O_2_ except that fluorescence intensity was higher in infected cells. Changes in nuclear morphology such as nuclear fragmentation and nuclear condensation were also observed (figure 2D). These results suggest that the rotavirus infection induced changes in cell membrane permeability of Sp2/0-Ag14 cells.

To further study the changes induced in the cell membrane by rotavirus infection, annexin V conjugated to Alexa Fluor 568 was used for the detection of phosphatidylserine externalization to the plasma membrane, an early apoptotic signal. Sp2/0-Ag14 cells were separately infected with the 5 rotavirus isolates indicated above using a MOI of 0.8. After 12 hpi, cells were found to be positive to annexin V fluorescence signals at percentages of 67.5%, 59.5%, 49%, 41.5%, and 36.5 % for rotavirus isolates WTEW, ECwt-O, WWM, TRUYO, and Wt1-5, respectively (figure 2E). The fluorescence pattern of rotavirus-infected cells was similar to that exhibited by H_2_O_2_-treated cells, except that a higher percentage of fluorescent cells were observed in the control counterpart (figure 2F). These images suggest that rotavirus infection can induce apoptotic signals in Sp2/0-Ag14 cells.

### Genotoxic and apoptotic effects induced by rotavirus infection

**Figure 3:**
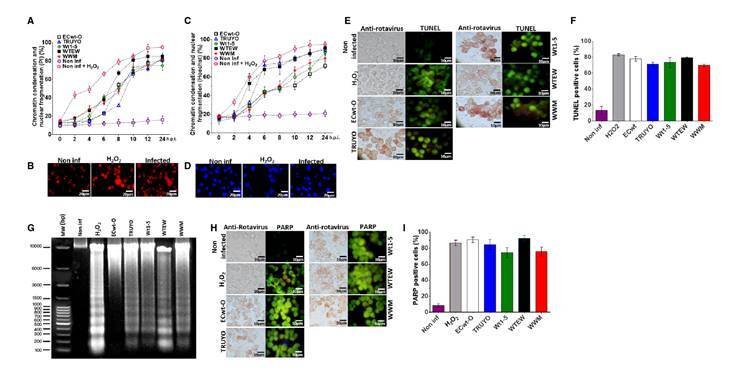
Genotoxic and apoptotic effects induced by rotavirus infection. Sp2/0-Ag14 cells were separately infected with rotavirus isolates ECwt-O, TRUYO, Wt1-5, WTEW, or WWM at MOI 0.8 each. Non-infected and H_2_O_2_-treated cells were used as negative and positive controls, respectively. A. Cell aliquots were harvested at the indicated times, stained with PI, and analyzed by epifluorescence microscopy. The percentages of cells showing chromatin condensation and nuclear fragmentation are indicated. B. Representative images of PI staining at 12 hpi from the cells analyzed in A. C. Cells were analyzed in terms of percentages as indicated in A, except that they were stained with Hoechst. D. Representative images of cells at 12 hpi exhibiting chromatin condensation and nuclear fragmentation. E. Cells at 12 hpi were tested for rotavirus structural antigen and DNA fragmentation (TUNEL labeling). Representative images are shown. F. Mean percentages of TUNEL positive cells are shown for each virus isolate tested in C. G. DNA fragmentation assay in agarose gel (1%) stained with SyBR Safe DNA^™^ gel stain is shown for cells infected with each virus isolate at 12 hpi. H. Representative images of cells showing rotavirus structural proteins in immunocytochemistry assay and PARP-1 expression in immunofluorescence assay at 12 hpi are shown for the virus isolates indicated. I. The mean percentages of cells showing PARP-1 fluorescent signals are shown for the virus isolates tested in H.

To investigate further apoptotic signals induced by rotavirus infection in Sp2/0-Ag14 cells, assays for testing DNA changes after infection were conducted using Hoechst or propidium iodide staining. The results showed that infected cells examined every 2 h until 24 hpi underwent progressive chromatin condensation and nuclear fragmentation throughout the period examined. In cells assayed with propidium iodide, signals of chromatin condensation and nuclear fragmentation were observed from 6 hpi and were detected in 20 to 40% of the cells, where higher percentages corresponded to cells infected with rotavirus isolates WTEW, WWM, and Wt1-5. After 10 hpi, the percentages of cells showing chromatin condensation and nuclear fragmentation reached 75 to 80% for all rotavirus isolates tested (figure 3A).

Staining assays using propidium iodide showed both chromatin condensation and nuclear fragmentation in rotavirus infected cells and in positive control cells that had been treated with H_2_O_2_. In contrast, these signals were absent in non-infected control cells (figure 3B). These results were confirmed after Hoechst staining (figure 3D). Representative images in figure 3B and 3D are from assays with the isolate WTEW recorded at 24 hpi.

In assays conducted using Hoechst, chromatin condensation and nuclear fragmentation were observed at 24 hpi in 90%, 88%, 82%, 78%, and 70% of cells infected with rotavirus isolates WTEW, TRUYO, WT1-5, WWM, and ECwt, respectively (figure 3C). In assays conducted with Hoechst, chromatin condensation and nuclear fragmentation affecting 18 to 32% of the cells were visualized from 4 hpi. At this time, slightly higher percentages of chromatin condensation and nuclear fragmentation were observed in cells infected with rotavirus isolates WTEW, WWM, and Wt1-5. The proportion of cells exhibiting chromatin condensation and nuclear fragmentation was increased from 6 hpi. for WTEW and TRUYO-infected cells reaching their highest percentages (75% and 88%, respectively) after 10 hpi. The percentages of cells showing chromatin condensation and nuclear fragmentation for the remaining rotavirus isolates tested also increased from 6 hpi, except that their percentages were slightly lower than those reached for isolates WTEW, WWM, and Wt1-5 after 10 hpi (figure 3C). These results suggest that infection of SP2/0-Ag14 cells with most of the rotavirus isolates studied can induce detectable apoptotic signals as early as 4 hpi.

A TUNEL assay was also used to assess DNA fragmentation after rotavirus infection of Sp2/0-Ag14 cells. This nick-end labeling method was followed in the same samples used in the immunocytochemistry assay to detect rotavirus structural antigens. A correlation was found between rotavirus- infected cells and TUNEL-positive ones. Fluorescent signals from TUNEL- positive cells showed a similar pattern to that of H_2_O_2_-treated control cells (figure 3E). The percentage of TUNEL-positive cells observed in infected cells was higher than 70% for the rotavirus isolates examined at 12 hpi (figure 3F). Detection of rotavirus-induced DNA fragmentation was further confirmed by agarose gel electrophoresis. This analysis indicated that DNA fragmentation is significantly associated with rotavirus infection and the rotavirus isolate WWM seemed to induce higher DNA fragmentation generating fragments with sizes lower than 300 bp (figure 3G).

PARP-1 (113 kDa) is a eukaryote constitutive factor implicated in DNA damage surveillance to deal with DNA strand breaks produced by both exogenous and endogenous genotoxic agents ^41^. Drawing on the fact that PARP-1 is activated by DNA single-strand breaks generated by some genotoxic agents and then cleaved into 89 and 24 kDa fragments during apoptosis, rotavirus-infected Sp2/0-Ag14 cells were assayed for their reactivity to a specific antibody to cleaved PARP-1. Fluorescent intensity associated with cleaved PARP-1 reactive cells was significantly higher in rotavirus- infected cells and fluorescent cells were correlated with those being positive to rotavirus antigen (figure 3H). The fluorescent pattern of cleaved PARP-1-positive cells was similar to that observed in H_2_O_2_-treated control cells, except that fluorescent intensity was higher in infected cells. The proportion of infected cells showing positive fluorescent signals for cleaved PARP-1 ranged from 72 to 91% for all the rotavirus isolates studied (figure 3I).

### Expression of cell surface proteins in Sp2/0-Ag14 cells

**Figure 4:**
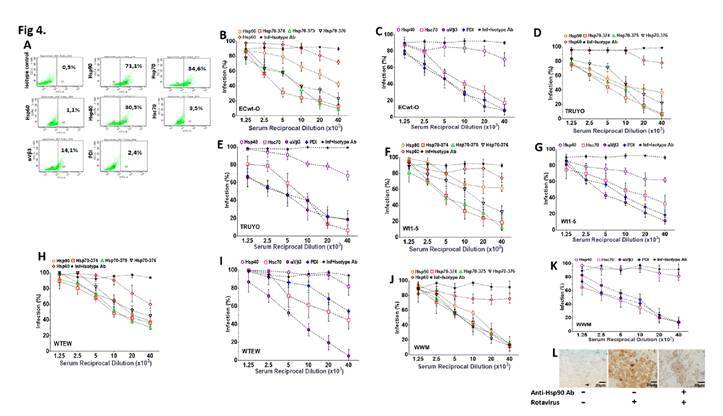
Inhibition of rotavirus infection by antibodies against cell surface proteins. A. Dot plots of fluorescent events in Sp2/0-Ag cells labeled by FITC-conjugated antibodies to the indicted cell surface proteins. Positive cells were selected by gating FITC-labelled cells in a FITC vs side scatter plot. B-K. Sp2/0-Ag cells were incubated with serial dilutions of antibodies against the indicated cell surface proteins or the indicated synthetic peptides. Cells were separately challenged with rotavirus isolates ECwt-O (B, C), TRUYO (D, E), Wt1-5 (F, G), WTEW (H, I) or WWM (J, K) at MOI 0.8 each. Percentage of infection Inhibition at 12 hpi in terms of cells positive to rotavirus structural antigens was expressed relative to the corresponding rotavirus-infected control cells that had not been antibody-treated. Infected and non-infected cells treated with unrelated isotype-matched antibodies were used as a control. Percentages were normalized to those of the infected cells that had been treated with isotype antibody. L. Representative images from an immunochemistry assay of cells non-infected (left panel), infected with virus isolate WTEW (central panel), and pre-treated with hyperimmune antiserum (reciprocal dilution 40 x 103) against Hsp90 (right panel) before infection with isolate WTEW.

Cell surface Hsc70 and integrins such as αVβ3 have been proposed to participate in rotavirus entry into the host cell ^33^^-^^35^. To test for the presence of these proteins on the cell surface of the Sp2/0-Ag14 cells and that of additional cell surface proteins induced by heat shock, they were subjected to epifluorescence and flow cytometry analysis using antibodies against Hsp40, Hsp60, Hsp70, Hsp90, rHsc70, rPDI, and integrin β3. The flow cytometry analysis showed a differential cell surface expression for the proteins studied. Hsp90, Hsp70, Hsp60, Hsp40, Hsc70, integrin β3, and PDI were expressed in 71.1%, 34.6%, 1.1%, 30,5%, 3.5%, 14.1, and 2.4% of the cells tested, respectively, when rabbit hyperimmune sera against HSPs, PDI, and integrin β3 were used as the source of primary antibodies (figure 4A).

### Inhibition of rotavirus infection by antibodies to cell surface proteins

Some cell surface proteins such as HSP, PD**I,** and integrin αVβ3 are markers of tumor progression and aggressiveness ^28^^,^^42^^-^^46^. Hsc70, PDI, and integrin αVβ3 have been proposed as cell surface receptors for rotavirus infection of non-tumor cells ^33^^-^^35^^,^^37^^,^^47^. In this context, we analyzed whether HSP, PDI, and integrin β3 were involved in the entry of rotavirus isolates Wt1- 5, ECwt-O, TRUYO, WWM, and WTEW into Sp2/0-Ag14 cells. To do so, cells were pre-treated with hyperimmune serum raised against synthetic peptides corresponding to specific regions of Hsp90, Hsp70, Hsp60, or Hsp40 or against rHsc70, rPDI, or integrin β3.

Following infection of antibody-pretreated cells, the percentage of infected cells at 12 hpi for all the rotavirus isolates tested was reduced by antibodies directed to Hsp90, Hsp70 (374, 375 and 376), Hsc70, PDI, and integrin β3 whereas no effect was observed in the case of antibodies to Hsp60 and Hsp40 (figures 4B-K).

Except for antibodies against Hsp60 and Hsp40, antibody pretreatment of cells decreased virus infection in a dose-dependent manner. In contrast, pretreatment of cells with the unrelated antibodies against potato virus Y did not affect rotavirus infection. Virus infection in the absence of cell antibody pretreatment was scored as 100%.

Representative images of immunochemistry assays of infected and non- infected control cells with or without antibody treatment are shown in figure 4L**.** These results suggest that cellular proteins Hsp60 and Hsp40 are not involved in the rotavirus entry into Sp2/0-Ag14 cells.

### Changes in PDI and HSP expression after rotavirus infection

**Figure 5:**
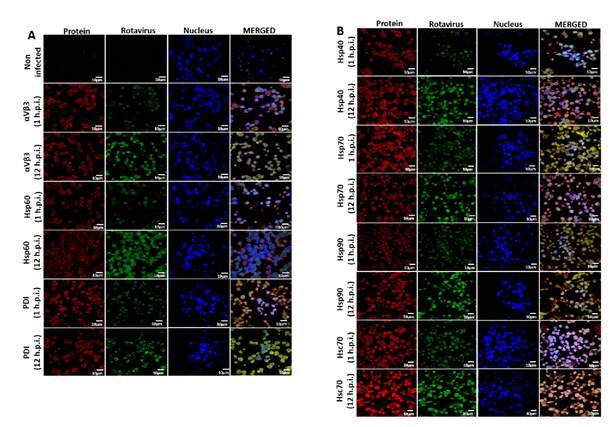
Effect of ECwt-O infection on the expression of cell surface proteins in Sp2/o-Ag cells. Cells were inoculated with ECwt-O at MOI 2. Confocal microscopy was used to determine expression changes of the indicted cellular proteins at 1 and 12 hpi. Alexa fluor 568 (red) and FITC (green)-labelled secondary antibodies were used to visualize cellular proteins and rotavirus structural proteins, respectively. Nuclei were counterstained with DAPI (blue). Cells were analyzed using a confocal microscope (Nikon C-1) and analyzed using the EZ-C1, ver. 3.90, software.

Some chaperone proteins including HSP change their expression pattern during the viral infection process ^48^^-^^51^. We wanted to study whether ECwt-O infection of Sp2/0-Ag14 cells was able to induce changes in the expression of Hsp90, Hsp70, Hsp60, Hsp40, Hsc70, PDI, or integrin β3. After confocal microscopy analysis of permeabilized infected cells using commercial antibodies against these cellular proteins, the fluorescence intensity of Hsc70, Hsp40, and Hsp90 increased at 12 hpi compared to that observed at 1 hpi, whereas only a moderate increase was detected for PDI and integrin β3 and no changes were registered in the fluorescence signals for Hsp70 (figure 5). In the case of Hsp60, the viral infection appeared to cause its redistribution (figure 5). Besides, Hsc70 were found to overlap with structural viral antigens at 12 hpi but not at 1 hpi. Hsp40 and Hsp90 showed only a faint overlapped signal with viral antigens. A faint overlapped signal for PDI and viral antigens was detected only at 1 hpi (figure 5).

### Discussion

Most viruses can cause the death of the host cell by interacting with the cell death machinery ^52^, and apoptosis is one of such mechanisms. Viruses can trigger apoptosis of infected cells through a variety of molecular events.

The present study attempted to assess the death mechanisms associated with rotavirus infection of Sp2/0-Ag14 cells as an approach to understand the virus-induced death of tumor cells. First of all, rotavirus isolates ECwt-O, WTEW, TRUYO, Wt1-5, and WWM were able to replicate in Sp2/0-Ag14 cells as newly synthesized viral antigens were progressively accumulated from 2 to 12 hpi in terms of the percentage of cells being positive to viral antigens. Moreover, supernatant and cell lysate fractions from rotavirus-infected Sp2/0-Ag14 cells harvested at different post-infection times were able to infect naïve cells, which suggests that mature infectious virions can be rescued from virus- producing Sp2/0-Ag14 cells. Interestingly, all the rotavirus isolates studied were unable to replicate in L929 mouse fibroblasts and human PBMC.

Regarding that infection of Sp2/0-Ag14 cells with rotavirus isolates ECwt-O, WTEW, TRUYO, WT1-5, or WWM-induced permeability changes and early apoptotic signals in the cell membrane, besides chromatin condensation, nuclear fragmentation, and DNA fragmentation, it is plausible to suggest that rotavirus is inducing an apoptotic response in infected cells. However, rotavirus-infected Sp2/0-Ag14 cells appear to undergo lysis rather than fragmentation into apoptotic bodies. Rotaviruses have been reported to induce apoptosis in Caco-2 cells ^53^ and oncosis in MA104 cells ^54^ where virus infection affects cell membrane integrity without inducing DNA fragmentation or formation of apoptotic bodies. On the other hand, rotavirus nonstructural protein NSP1 has been found to suppress apoptotic signaling during the first 6 h post-infection to favor cell survival ^55^.

Interaction between viruses and their host cells is characterized by interactions between cell surface receptors and virus structural proteins that facilitate entry. Once inside the host cell, many virus-encoded proteins can modify cellular transcription and translation patterns to favor virus multiplication.

In this regard, we wanted to study the implication of some cell surface proteins in the infection of Sp2/0-Ag14 cells by rotavirus isolates and the changes in the expression of these cellular proteins induced by the viral infection. Cell surface Hsc70, PDI, and integrin αVβ3 have been shown to interact with rotavirus structural proteins during entry into MA104 or Caco-2 cells ^40^^,^^56^^,^^57^. These cellular proteins, in addition to some HSP, have also been associated with cell malignancy ^58^^,^^59^.

Here, we found through flow cytometry and epifluorescence analysis that Sp2/0-Ag14 cells express moderate levels of Hsp90 and Hsp70 on their cell surface while Hsp60, Hsp40, Hsc70, PDI, and integrin β3 are expressed at low levels. These results prompted us to examine the implication of these proteins in the infection of cells Sp2/0-Ag14 cells by rotavirus isolates such as that demonstrated with Hsc70, PDI, and integrin β3 in MA104 and Caco- 2 cells and mouse enterocytes ^35^^,^^56^^,^^60^. Pre-incubation of cells with antibodies to Hsp90, Hsp70, Hsc70, PDI, or integrin β3 resulted in decreased viral infection suggesting that these cellular proteins are used to some extent by the rotavirus isolates studied. Since all cell antibody treatments were able to completely abolish virus infection, it is suggested that rotaviruses appear to have evolved to use alternative cell surface molecules as entry pathways ^36^.

Here we have shown that the cellular proteins reacting with their respective antibodies fall within the three main types of cell surface proteins required in the entry into the host cell ^36^. For instance, integrin β3 is a cell surface molecule that mediates rotavirus attachment to cell ^35^^,^^56^ while rotavirus entry needs chaperone activities and redox reactions performed by HSPs/Hsc70 ^33^^,^^61^^,^^62^ and PDI ^37^^,^^57^, respectively. In addition, specific inhibition of Hsp90 has been reported to decrease infection by human rotavirus strain KU and simian rotavirus strain SA11 through modulation of cellular signaling proteins ^61^.

Nevertheless, the implication of other cell surface proteins in rotavirus entry into Sp2/0-Ag14 cells cannot be discarded and further studies are needed. The present report highlights the role of new HSP (Hsp70, Hsp60, and Hsp40) in rotavirus infection, particularly in infection caused by reassortant rotavirus isolates adapted to tumoral cells ^36^. The expression of cellular HSP has been found to change during viral infections ^48^^-^^51^. Although host mRNA synthesis and translation were not measured in the present work, the net accumulation of the cellular proteins studied was determined at 12 hpi and compared to their expression levels at 1 hpi. Confocal analysis indicated a virus-induced differential accumulation of the cellular proteins studied where Hsc70, Hsp40, and Hsp90 accumulation was higher than that observed for PDI while a moderate change was found for PDI and no change was detected for Hsp70.

Although a redistribution of Hsp60 appeared to be caused by the viral infection at 12 hpi, we cannot say conclusively whether this redistribution involved either or both the cytoplasm or the cell membrane. However, the merged image seems to suggest that co-localization of the viral antigen and Hsp60 did not occur. Also, an apparent accumulation of Hsp60 in the cell membrane appeared to be induced by the viral infection but further assays should be conducted to confirm this apparent change in the Hsp60 redistribution. Interestingly, Hsc70 was found to overlap with viral antigens at 12 hpi. This result suggests that Hsc70 might play a role during the late stages of viral infection. However, only faint overlapping signals of viral antigens with Hsp40 and Hsp90 were detected at the same post-infection time suggesting that a minor direct role is played by these proteins at this post-infection time.

Co-localization is used in fluorescence microscopy analysis to detect protein interactions based on the signals emitted by fluorescently labeled protein species. However, interactions derived from co-localization analyses do not necessarily mean direct interaction between two proteins whose confirmation sometimes requires the use of fluorescence resonance energy transfer (FRET). In the same line of thought, although fluorescence overlapping does not necessarily mean co-localization of two molecules, in principle it may suggest an interaction. Hence, with this precaution, we can speculate that the overlapping of fluorescent signals from PDI and rotavirus structural antigens in permeabilized cells suggests a probable interaction of these proteins during the early stages of rotavirus infection.

The analysis of the fluorescent signals from integrin β3 and Hsc70 also suggests that these cellular proteins might interact with virion antigens during the early stages of viral life cycle. Fluorescent signals from Hsp70 and Hsp90, although in low percentage, seemed to be overlapped with those of viral antigens. Interaction of rotavirus with cellular chaperone Hsp90 has been reported during the formation of functional rotavirus nonstructural protein NSP3 (102). The strong fluorescent overlapping between Hsp70 and ECwt-O antigens at 12 hpi would suggest that this cellular protein plays some role at the late stages of the viral cycle. However, the overlapping fluorescent signal analysis needs to be confirmed at least by a confocal microscope analysis.

The ability of the rotavirus isolates here tested to replicate and cause cell death of Sp2/0-Ag14 cells through mechanisms that are compatible with virus- induced apoptosis makes these viral isolates potential candidates to be used as oncolytic agents.

## References

[1] Siegel RL, Miller KD, Jemal A (2017). Cancer Statistics, 2017. CA Cancer J Clin.

[2] Townsend N, Wilson L, Bhatnagar P, Wickramasinghe K, Rayner M, Nichols M (2016). Cardiovascular disease in Europe: Epidemiological update 2016. Eur Heart J.

[3] Guo W, Chen W, Yu W, Huang W, Deng W (2013). Small interfering RNA-based molecular therapy of cancers. Chin J Cancer.

[4] Garzón R, Marcucci G, Croce CM (2010). Targeting microRNAs in cancer: Rationale, strategies and challenges. Nat Rev Drug Discov.

[5] Morton SW, Lee MJ, Deng ZJ, Dreaden EC, Siouve E, Shopsowitz KE (2014). A nanoparticle- based combination chemotherapy delivery system for enhanced tumor killing by dynamic rewiring of signaling pathways. Sci Signal.

[6] Atherton MJ, Lichty BD (2013). Evolution of oncolytic viruses: Novel strategies for cancer treatment. Immunotherapy.

[7] Negrini S, Gorgoulis VG, Halazonetis TD (2010). Genomic instability--an evolving hallmark of cancer. Nat Rev Mol Cell Biol.

[8] McGranahan N, Swanton C (2015). Biological and therapeutic impact of intratumor heterogeneity in cancer evolution. Cancer Cell.

[9] Merajver S, Phadke S, Soellner M (2017). Conquering the challenges of genotypic and phenotypic tumor heterogeneity to realize the promise of personalized cancer therapy: The role of academia. Trans Am Clin Climatol Assoc.

[10] Berghella AM, Contasta I, Lattanzio R, Di Gregorio G, Campitelli I, Silvino M (2017). The role of gender-specific cytokine pathways as drug targets and gender-specific biomarkers in personalized cancer therapy. Curr Drug Targets.

[11] Hainaut P, Plymoth A (2013). Targeting the hallmarks of cancer: Towards a rational approach to next-generation cancer therapy. Curr Opin Oncol.

[12] Singh PK, Doley J, Kumar GR, Sahoo AP, Tiwari AK (2012). Oncolytic viruses and their specific targeting to tumour cells. Indian J Med Res.

[13] Howells A, Marelli G, Lemoine NR, Wang Y (2017). Oncolytic viruses-interaction of virus and tumor cells in the battle to eliminate cancer. Front Oncol.

[14] Kaufman HL, Kohlhapp FJ, Zloza A (2015). Oncolytic viruses: A new class of immunotherapy drugs. Nat Rev Drug Discov.

[15] Farassati F, Yang AD, Lee PW (2001). Oncogenes in Ras signalling pathway dictate host-cell permissiveness to herpes simplex virus 1. Nat Cell Biol.

[16] Garant KA, Shmulevitz M, Pan L, Daigle RM, Ahn DG, Gujar SA (2016). Oncolytic reovirus induces intracellular redistribution of Ras to promote apoptosis and progeny virus release. Oncogene.

[17] Kohlhapp FJ, Kaufman HL (2016). Molecular pathways: Mechanism of action for talimogene laherparepvec, a new oncolytic virus immunotherapy. Clin Cancer Res.

[18] Ebrahimi S, Ghorbani E, Khazaei M, Avan A, Ryzhikov M, Azadmanesh K (2017). Interferon- mediated tumor resistance to oncolytic virotherapy. J Cell Biochem.

[19] Vaha-Koskela M, Hinkkanen A (2014). Tumor restrictions to oncolytic virus. Biomedicines.

[20] Okamoto J, Mikami I, Tominaga Y, Kuchenbecker KM, Lin YC, Bravo DT (2008). Inhibition of Hsp90 leads to cell cycle arrest and apoptosis in human malignant pleural mesothelioma. J Thorac Oncol.

[21] Truman AW, Kristjansdottir K, Wolfgeher D, Hasin N, Polier S, Zhang H (2012). CDK- dependent Hsp70 phosphorylation controls G1 cyclin abundance and cell-cycle progression. Cell.

[22] Diehl JA, Yang W, Rimerman RA, Xiao H, Emili A (2003). Hsc70 regulates accumulation of cyclin D1 and cyclin D1-dependent protein kinase. Mol Cell Biol.

[23] Lanneau D, Brunet M, Frisan E, Solary E, Fontenay M, Garrido C (2008). Heat shock proteins: Essential proteins for apoptosis regulation. J Cell Mol Med.

[24] Basu S, Srivastava PK (2000). Heat shock proteins: The fountainhead of innate and adaptive immune responses. Cell Stress Chaperones.

[25] Binder RJ (2014). Functions of heat shock proteins in pathways of the innate and adaptive immune system. J Immunol.

[26] Lianos GD, Alexiou GA, Mangano A, Rausei S, Boni L, Dionigi G (2015). The role of heat shock proteins in cancer. Cancer Lett.

[27] Murphy ME (2013). The HSP70 family and cancer. Carcinogenesis.

[28] Lee E, Lee DH (2017). Emerging roles of protein disulfide isomerase in cancer. BMB Rep.

[29] Liu Z, Wang F, Chen X (2008). Integrin alpha(v)beta(3)-targeted cancer therapy. Drug Dev Res.

[30] Mangurten AB, Brader KR, Loos BM, Lee E, Quiroga AI, Bathori J (1997). Hsp70 and Hsc70 are preferentially expressed in differentiated epithelial cells in normal human endometrium and ectocervix. Cell Stress Chaperones.

[31] Essex DW, Chen K, Swiatkowska M (1995). Localization of protein disulfide isomerase to the external surface of the platelet plasma membrane. Blood.

[32] Calderwood SK, Gong J (2016). Heat shock proteins promote cancer: It’s a protection racket. Trends Biochem Sci.

[33] Guerrero CA, Bouyssounade D, Zarate S, Isa P, López T, Espinosa R (2002). Heat shock cognate protein 70 is involved in rotavirus cell entry. J Virol.

[34] Calderón MN, Guerrero CA, Acosta O, López S, Arias CF (2012). Inhibiting rotavirus infection by membrane-impermeant thiol/disulfide exchange blockers and antibodies against protein disulfide isomerase. Intervirology.

[35] Guerrero CA, Méndez E, Zarate S, Isa P, López S, Arias CF (2000). Integrin alpha(v)beta(3) mediates rotavirus cell entry. Proc Natl Acad Sci USA.

[36] Guerrero CA, Guerrero RA, Silva E, Acosta O, Barreto E (2016). Experimental adaptation of rotaviruses to tumor cell lines. PLoS One.

[37] Calderón MN, Guzmán F, Acosta O, Guerrero CA (2012). Rotavirus VP4 and VP7-derived synthetic peptides as potential substrates of protein disulfide isomerase lead to inhibition of rotavirus infection. Int J Pept Res Ther.

[38] Prieto I, Hervas-Stubbs S, García-Granero M, Berasain C, Riezu-Boj JI, Lasarte JJ (1995). Simple strategy to induce antibodies of distinct specificity: Application to the mapping of gp120 and inhibition of HIV-1 infectivity. Eur J Immunol.

[39] Arnold M, Patton JT, McDonald SM (2009). Culturing, storage, and quantification of rotaviruses. Curr Protoc Microbiol.

[40] Moreno LY, Guerrero CA, Acosta O (2016). Protein disulfide isomerase and heat shock cognate protein 70 interactions with rotavirus structural proteins using their purified recombinant versions. Revista Colombiana de Biotecnología.

[41] Shall S, de Murcia G (2000). Poly(ADP-ribose) polymerase-1: What have we learned from the deficient mouse model?. Mutat Res.

[42] Ciocca DR, Arrigo AP, Calderwood SK (2013). Heat shock proteins and heat shock factor 1 in carcinogenesis and tumor development: An update. Arch Toxicol.

[43] Samanta S, Tamura S, Dubeau L, Mhawech-Fauceglia P, Miyagi Y, Kato H (2017). Expression of protein disulfide isomerase family members correlates with tumor progression and patient survival in ovarian cancer. Oncotarget.

[44] Lorger M, Krueger JS, O’Neal M, Staflin K, Felding-Habermann B (2009). Activation of tumor cell integrin alphavbeta3 controls angiogenesis and metastatic growth in the brain. Proc Natl Acad Sci USA.

[45] Havaki S, Kouloukoussa M, Amawi K, Drosos Y, Arvanitis LD, Goutas N (2007). Altered expression pattern of integrin alphavbeta3 correlates with actin cytoskeleton in primary cultures of human breast cancer. Cancer Cell Int.

[46] Bai SY, Xu N, Chen C, Song YL, Hu J, Bai CX (2015). Integrin alphavbeta5 as a biomarker for the assessment of non-small cell lung cancer metastasis and overall survival. Clin Respir J.

[47] Gualtero DF, Guzmán F, Acosta O (2007). Guerrero CA. Amino acid domains 280-297 of VP6 and 531-554 of VP4 are implicated in heat shock cognate protein hsc70-mediated rotavirus infection. Arch Virol.

[48] Kim MY, Oglesbee M (2012). Virus-heat shock protein interaction and a novel axis for innate antiviral immunity. Cells.

[49] Khandjian EW, Turler H (1983). Simian virus 40 and polyoma virus induce synthesis of heat shock proteins in permissive cells. Mol Cell Biol.

[50] Wu BJ, Hurst HC, Jones NC, Morimoto RI (1986). The E1A 13S product of adenovirus 5 activates transcription of the cellular human HSP70 gene. Mol Cell Biol.

[51] Phillips B, Abravaya K, Morimoto RI (1991). Analysis of the specificity and mechanism of transcriptional activation of the human hsp70 gene during infection by DNA viruses. J Virol.

[52] Kaminskyy V, Zhivotovsky B (2010). To kill or be killed: How viruses interact with the cell death machinery. J Intern Med.

[53] Bautista D, Rodríguez LS, Franco MA, Ángel J, Barreto A (2015). Caco-2 cells infected with rotavirus release extracellular vesicles that express markers of apoptotic bodies and exosomes. Cell Stress Chaperones.

[54] Pérez JF, Chemello ME, Liprandi F, Ruiz MC, Michelangeli F (1998). Oncosis in MA104 cells is induced by rotavirus infection through an increase in intracellular Ca2+ concentration. Virology.

[55] Bagchi P, Dutta D, Chattopadhyay S, Mukherjee A, Halder UC, Sarkar S (2010). Rotavirus nonstructural protein 1 suppresses virus-induced cellular apoptosis to facilitate viral growth by activating the cell survival pathways during early stages of infection. J Virol.

[56] Zarate S, Romero P, Espinosa R, Arias CF, López S (2004). VP7 mediates the interaction of rotaviruses with integrin alphavbeta3 through a novel integrin-binding site. J Virol.

[57] Calderón MN, Guerrero CA, Domínguez Y, Garzón E, Barreto SM, Acosta O (2011). Interacción de rotavirus con la proteína disulfuro-isomerasa *in vitro* y en sistemas celulares.

[58] Wang J, Cui S, Zhang X, Wu Y, Tang H (2013). High expression of heat shock protein 90 is associated with tumor aggressiveness and poor prognosis in patients with advanced gastric cancer. PLoS One.

[59] Zhang S, Hu Y, Huang Y, Xu H, Wu G, Dai H (2015). Heat shock protein 27 promotes cell proliferation through activator protein-1 in lung cancer. Oncol Lett.

[60] Isa P, Sánchez-Alemán MA, López S, Arias CF (2009). Dissecting the role of integrin subunits alpha 2 and beta 3 in rotavirus cell entry by RNA silencing. Virus Res.

[61] Dutta D, Chattopadhyay S, Bagchi P, Halder UC, Nandi S, Mukherjee A (2011). Active participation of cellular chaperone Hsp90 in regulating the function of rotavirus nonstructural protein 3 (NSP3). J Biol Chem.

[62] Dutta D, Bagchi P, Chatterjee A, Nayak MK, Mukherjee A, Chattopadhyay S (2009). The molecular chaperone heat shock protein-90 positively regulates rotavirus infectionx. Virology.

